# Impact research of pain nursing combined with hospice care on quality of life for patients with advanced lung cancer

**DOI:** 10.1097/MD.0000000000037687

**Published:** 2024-05-31

**Authors:** Ting Yuan, Yan Zhou, Ting Wang, Yan Li, Yanli Wang

**Affiliations:** a Department of Thoracic Oncology, Shanxi Bethune Hospital, The Third Clinical Medical School of Shanxi Medical University, Taiyuan, Shanxi, China.

**Keywords:** advanced lung cancer, hospice care, pain nursing, quality of life

## Abstract

This study aims to evaluate the impact of integrating pain nursing with hospice care on the quality of life among patients with advanced lung cancer. This study involving 60 advanced lung cancer patients admitted from January 2022 to January 2023. Participants were randomly assigned to 2 groups: the observation group received a combination of pain nursing and hospice care, while the control group received standard nursing care. The study assessed changes in the numeric rating scale for pain, self-rating anxiety scale (SAS), self-rating depression scale (SDS), cancer fatigue scale (CFS), death attitude, and various quality of life dimensions as measured by the Quality of Life Questionnaire-Core 30. Post-intervention, both groups exhibited reductions in numeric rating scale, SAS, SDS, and CFS scores compared to baseline, with more significant improvements observed in the observation group (*P* < .05). Additionally, post-intervention scores for death attitude and Quality of Life Questionnaire-Core 30 domains (physical, cognitive, social, role, and emotional functioning, as well as overall health) increased in both groups, with the observation group showing greater improvements than the control group (*P* < .05). The combination of pain nursing and hospice care significantly reduces pain, anxiety, and depression, decreases cancer-related fatigue, and improves the quality of life and death attitudes in patients with advanced lung cancer, highlighting the benefits of this integrative approach in palliative care settings.

## 1. Introduction

Lung cancer, a prevalent malignancy, often presents with nonspecific early symptoms, leading to a majority of diagnoses at advanced stages, significantly impacting treatment efficacy. Patients with advanced lung cancer face considerable physical and psychological challenges, including fatigue, dyspnea, sleep disturbances, and persistent diarrhea, compounded by anxiety, fear, guilt, and concerns for their family and future.^[1]^ Despite a slight improvement in the 3-year relative survival rate for advanced lung cancer from 21% to 31% since 2011, the overall prognosis remains poor. In 2022, an estimated 609,360 cancer-related deaths are expected in the United States, with lung cancer accounting for nearly 350 daily fatalities, maintaining its position as the leading cause of cancer mortality.^[2]^ Given this context, the provision of palliative care, emphasizing compassion and social support, holds significant societal importance, offering a means to alleviate the suffering of those with terminal illnesses and enhance their quality of life.

Recent advancements in medical philosophy have increasingly focused on mitigating the suffering of terminal patients and guiding them towards a dignified acceptance of death. Hospice care, a model of comprehensive and compassionate treatment, offers personalized and evidence-based care to individuals with terminal conditions such as advanced cancer, AIDS, and other chronic illnesses.^[3–5]^ It addresses not only the physical discomforts but also the psychological, social, and spiritual challenges faced by patients and their families, aiming to enhance their quality of life. Hospice care enables individuals to live their remaining days in comfort, peace, and dignity, fostering a serene acceptance of life’s end. Research supports the efficacy of hospice care in significantly improving the emotional well-being and overall quality of life for patients facing advanced stages of cancer and other life-limiting diseases, underscoring its critical role in end-of-life care.^[6,7]^

Pain is a prevalent and distressing symptom among cancer patients, particularly those with advanced lung cancer, where research indicates that over 70% experience severe, often unbearable pain. This pain inflicts multidimensional suffering, encompassing physical agony, psychological distress, and spiritual despair, profoundly impacting their quality of life. The severity of this pain is such that it leads to suicidal ideation in at least 50% of those enduring intense discomfort, underscoring the critical need for effective pain management and holistic support in cancer care.^[8,9]^ Therefore, investigating pain management strategies for patients with advanced lung cancer is crucial for enhancing their quality of life and mental well-being. This study aims to explore the impact of integrative pain nursing and hospice care on the survival quality of individuals with advanced lung cancer, highlighting the importance of comprehensive care approaches in alleviating suffering and improving patient outcomes in terminal stages of the disease.

## 2. Methodologies

### 2.1. Study subjects

The study received approval from the Ethics Committee of Shanxi Bethune Hospital. Conducted as a single-center, randomized controlled trial, 60 patients with advanced lung cancer admitted to our hospital from January 2022 to January 2023 were randomly selected as participants. The target recipients of hospice care internationally are those patients who are in the terminal phase of their life, meaning that under the normal progression of the illness, the primary physician or the medical director responsible for the hospice care program has determined that the patient has a life expectancy of 6 months or less. Thus, inclusion criteria encompassed: (1) conformity with clinical diagnostic standards for lung cancer; (2) TNM staging of III–IV; (3) anticipated survival of more than 1 month but less than or equal to 6 months; (4) age over 18 years; (5) preserved cognitive function and communicative capacity, with the ability to accurately convey feelings and needs. Exclusion criteria included: (1) presence of other malignant tumors or critical illnesses; (2) severe consciousness disorders or psychiatric conditions; (3) employment in medical-related fields; (4) significant psychological stress from external factors during the study. All participants provided informed consent, and the hospital’s ethics committee sanctioned the study.

### 2.2. Nursing program

#### 2.2.1. Control group.

Patients in the control group were adopted the conventional nursing, inclusions: ① pain nursing: recording time, degree and the duration on the onset of pain, and offering patients analgesic drugs as doctor prescribed. ② Psychological intervention: guiding patients to recall the happy events in their past life, encouraging patients to follow doctor’s instructions in their medication. ③ Dietary intervention: according to patients’ condition, offering highly digestible food to patients and prohibiting them from eating stimulating food. ④ Family intervention: Informing the family about the patient’s condition, instructing the family to meet the patient’s reasonable needs and wishes in daily life as much as possible, at the same time, enlightening the patient to maintain a good mood. ⑤ Sleep intervention: assisting patients to establish a good daily routine and ensuring the sufficient sleep on them.

#### 2.2.2. Observation group.

The patients in the observation group received the pain nursing combined with hospice care, and the specific measures^[10,11]^ as following:

1. Pain nursing interventions included: ① music intervention: engaging with patients during pain-free periods to understand their personalities and musical preferences, and playing harmonious, soothing music they enjoy when they experience pain, to help stabilize their emotions. ② Somatic intervention: providing timely care for patients’ oral and skin health to enhance daily comfort, and instructing on breathing exercises to help manage pain through controlled breathing techniques. ③ Massage instruction: nurses perform a specific massage technique, using the thumb pads to massage from the right rib to the front of the abdomen for 3 to 10 minutes, followed by using the thenar eminence for an additional 5 to 10 minutes on the right back to alleviate pain. ④ Environmental intervention: placing books or ornaments that interest the patient in their room, encouraging engagement with these items as a distraction from disease and pain. ⑤ Pharmacological intervention: implementing the WHO’s three-step analgesic ladder for cancer pain management, which involves escalating the use of analgesics from non-opioids for mild pain, to opioids for moderate pain, and strong opioids for severe pain. The visual analogue scale is used to assess pain intensity and duration, ensuring analgesic administration aligns with medical prescriptions. The onset and duration of drug efficacy, along with changes in patient expressions post-administration, are meticulously recorded to enhance the rationality and effectiveness of pharmacological interventions.
2. Hospice care: ① information collection: communicating with patients and their families to learn about the patients’ interests and their knowledge on their disease and death, whether they can accept the missionary approach or not, etc, those procedures are aiming to formulate a basis for the scientific hospice care; ② Environmental intervention: choosing warm-colored ornaments to decorate the ward, placing greenery and the patient’s favorite items in the ward in order to enhance the sense of coziness at the ward; ③ psychological intervention: apprehending the psychological state of the patient, taking a reasonable way to answer their internal concerns, and providing guidance on the family members. The patient’s family members are instructed to avoid excessive sadness when taking care of the patient to ensure that the patient’s emotions are stable, at the same time, patiently asking the patient’s wishes and demands and trying to meet their reasonable needs; Discussing about the topic of death with the patient, listening carefully to the patient’s own experience and the expression from their heart on their success and disappointment, entering into the patient’s inner world with a sincere attitude and talking about the value of life and the law of human existence in the natural world; ④ Disease guidance: informing patients and their family of the specific state of an illness, the current treatment and the development of the disease. When informing the specific situation, closely observing the patient’s reaction and taking corresponding measures to calm the patient down timely; ⑤ life and death guidance: playing the documentary that refers to life origin and death for enhancing patients’ knowledge on life and death, reducing their fears on death, and guiding patients to face death correctly. ⑥ Daily intervention: based on the state of an illness on patients, developing a nutrition intervention plan to ensure sufficient nutrition and calorie intake for patients, guiding patients’ family members to meet the patient’s needs as much as possible in daily life, appropriately providing the foods that patients favored, and timely removing the patient’s oral secretion and food residue. Through the measures such as skin intervention and position intervention for improving patients’ comfort level; ⑦ the comfort and help on relatives: the relatives care-givers of patients should be equally enlightened and comforted. Helping their relatives to reduce the strong pressure on their spirits. Giving guidance on family members for the kindred-like nursing of patients. Nursing staff need to do the perspective taking with patients’ relatives, prompting their relatives to maintain a comparatively good mindset and cooperate with the hospital for completing various tasks for patients when the patients approaching their ends.

### 2.3. Observation indicators

#### 2.3.1. Numeric rating scale

The pain level of the patient was assessed by using numeric rating scale (NRS).^[12]^ A number 0 to 10 indicating the level of pain. A straight line was divided equally into 10 segments, and the level of pain was assessed in the order of 0 to 10 points with higher scores indicating higher levels of pain.

#### 2.3.2. Anxiety and depression

Self-rating anxiety scale (SAS)^[13]^ and self-rating depression scale (SDS)^[14]^ were used to assess the anxiety and depression of patients. The both of SAS and SDS have 20 items separately, each item corresponds to a score of 0 to 4, and the scores of each item were summed to get the total raw score, which was converted into the standard score. The critical value of the standard score is 50. The higher the score, the more serious the anxiety and depression.

#### 2.3.3. Cancer-caused fatigue

The cancer fatigue scale (CFS)^[15]^ was used to evaluate the level of cancer-caused fatigue in patients, which includes 3 dimensions of physical fatigue, emotional fatigue, and cognitive fatigue, with a total of 15 items. Each item is rated from 1 to 5 points. The higher the score, the severer the degree of fatigue on patients.

#### 2.3.4. Attitude on death

*Questionnaire of the attitude on death* was used to learn about patients’ attitudes on death,^[16]^ which is divided into 18 items and rated on a scale of 1 to 7. A score ≥ 5 indicating that they are able to accept death compliantly.

#### 2.3.5. Quality of life

Patients’ quality of life was assessed by the European organization for research and treatment of cancer quality of life questionnaire-C30,^[17]^ which consists of 5 functional scales among somatic function, cognitive function, social function, role function, and emotional function as well as a general health. Each of which is rated on a scale of 0–100. The higher scores indicating better quality of survival.

### 2.4. Statistical analysis

Data were analyzed and processed by SPSS23.0 software. Continuous variables such as age, NRS, SAS, SDS, CFS, the scale scores on death attitude, and QLQ-C30 scores were first tested for normality to confirm that they all approximately obeyed the normal distribution, and then evaluated by *t*-test or Welch test according to the results from the homogeneity of variance. Categorical variables such as gender, smoking history, and the type of lung cancer were expressed as (n, %) and were subjected to the chi-square test or rank-sum test. *P* < .05 indicating a statistically significant difference.

## 3. Result

### 3.1. Comparison of general information

This study encompassed 60 participants, divided equally into 2 groups: 30 in the observation group and 30 in the control group. Upon comparison, no significant disparities were found between the 2 groups regarding key demographic and clinical variables, including age, gender, smoking history, and lung cancer subtypes. The differences observed were not statistically significant, with a *P*-value > .05, indicating similarity in the baseline characteristics between the observation and control groups. Refer to Table 1 for detailed comparisons.

**Table 1:** Comparison of participants’ general information characteristics.

Item	Observation group (n = 30)	Control group (n = 30)	*t/X²*	*P*
Age (years)	66.31 ± 6.91	67.69 ± 6.39	0.801	.426
*Gender*			0.071	.790
Man	18 (60.00%)	19 (63.33%)		
Woman	12 (40.00%)	11 (36.67%)		
Smoking history	16 (53.33%)	14 (46.67%)	0.266	.605
*Types of lung cancer*			0.489	.783
Non-small cell lung cancer	16 (53.33%)	17 (56.67%)		
Adenocarcinoma	6 (20.00%)	4 (13.33%)		
Squamous carcinoma	8 (26.67%)	9 (30.00%)		

### 3.2. Comparison on NRS

**Figure 1.**
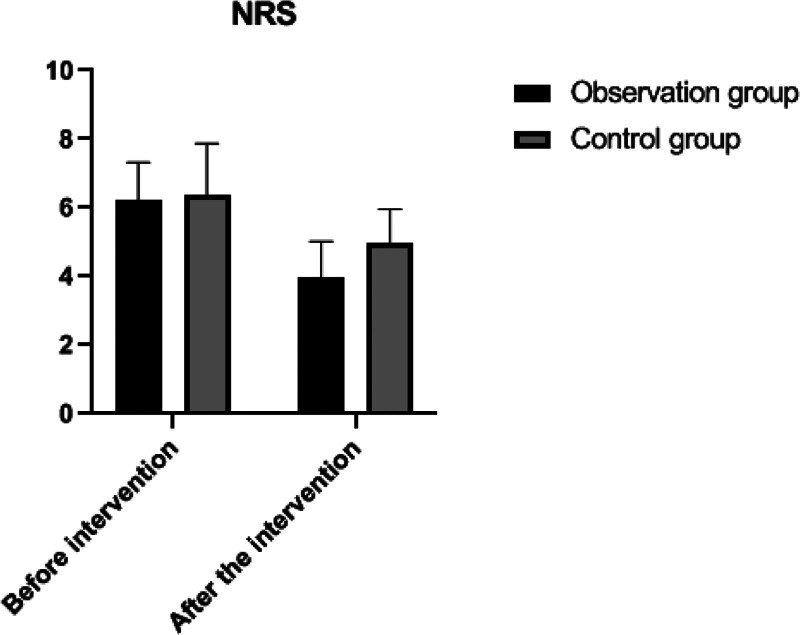
Changes on NRS in both groups. NRS = numeric rating scale.

Before the intervention, the NRS scores for pain were compared between the 2 groups, showing no statistically significant difference (*P* > .05). Following the intervention, the NRS scores in both groups decreased compared to their respective pre-intervention levels, with the observation group showing a more significant reduction in pain scores than the control group, a difference that was statistically significant (*P* < .05). This indicates that the intervention was effective in reducing pain, particularly in the observation group. Detailed data and comparisons are presented in Table 2 and illustrated in Figure 1.

**Table 2:** Comparison on NRS.

Groups	n	Pre-intervention	Post-intervention	*t*	*P*
Observation group	30	6.20 ± 1.11	3.98 ± 1.04	6.928	<.001
Control group	30	6.37 ± 1.50	4.96 ± 0.99	4.414	<.001
*t*		0.504	3.735		
*P*		0.616	<0.001		

NRS = numeric rating scale.

### 3.3. Comparison on anxiety and depression

**Figure 2.**
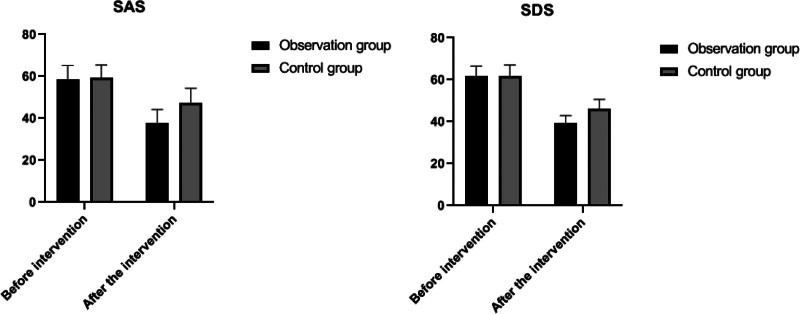
Change in SAS and SDS in both groups. SAS = self-rating anxiety scale, SDS = self-rating depression scale.

Before the intervention, the SAS and self-rating depression scale (SDS) scores were compared between the 2 groups, and no significant difference was observed (*P* > .05). Following the intervention, both SAS and SDS scores decreased in each group compared to their respective pre-intervention levels, indicating improvements in anxiety and depression symptoms. Notably, the reductions in SAS and SDS scores in the observation group were more significant than those in the control group, with this difference being statistically significant (*P* < .05). This suggests that the intervention was more effective in alleviating anxiety and depression in the observation group. Detailed results and comparisons are provided in Table 3 and depicted in Figure 2.

**Table 3:** Comparison on anxiety and depression.

Groups	n	SAS		SDS	
		Pre-intervention	Post-intervention	Pre-intervention	Post-intervention
Observation group	30	58.54 ± 6.72	37.64 ± 6.56*	61.60 ± 4.93	39.47 ± 3.45*
Control group	30	59.23 ± 6.21	47.31 ± 7.08*	61.98 ± 5.04	46.31 ± 4.32*
*t*		0.413	5.485	0.298	6.759
*P*		.680	<.001	.766	<.001

*Note*: Compared with the situation in pre-intervention.

* *P* < .05.

### 3.4. Comparison on cancer-caused fatigue and patients’ attitudes on death

**Figure 3.**
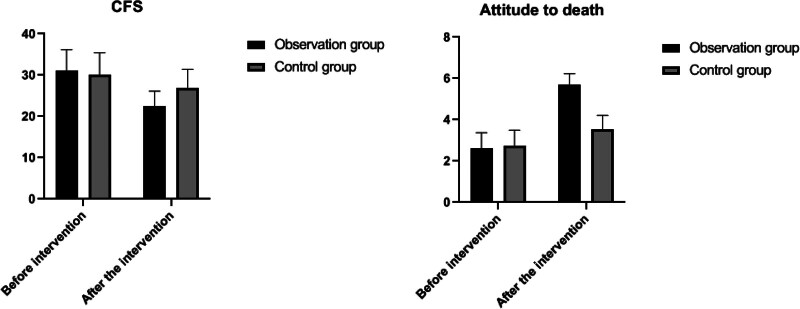
Changes in CFS and scores of death attitude in 2 groups. CFS = cancer fatigue scale.

Prior to the intervention, the cancer fatigue scale (CFS) scores and death attitude assessments for both groups were compared, revealing no significant differences (*P* > .05). Post-intervention, CFS scores in both groups decreased relative to their pre-intervention levels, indicating a reduction in fatigue. Additionally, post-intervention scores on death attitude were higher than pre-intervention scores within each group, suggesting an improved acceptance or perspective towards death (*P* < .05). Notably, the observation group exhibited lower CFS scores and higher death attitude scores compared to the control group after the intervention, indicating a statistically significant difference (*P* < .05). This implies that the intervention was more effective in reducing fatigue and positively influencing death attitudes in the observation group. These findings are detailed in Table 4 and illustrated in Figure 3.

**Table 4:** Comparison on cancer-caused fatigue and patients’ attitude on death

Groups	n	CFS		Attitude on death	
		Pre-intervention	Post-intervention	Pre-intervention	Post-intervention
Observation group	30	31.17 ± 4.98	22.50 ± 3.63*	2.61 ± 0.76	5.72 ± 0.51*
Control group	30	30.06 ± 5.37	26.97 ± 4.45*	2.75 ± 0.74	3.53 ± 0.68*
*t*		0.835	4.259	0.708	14.012
*P*		.407	<.001	.481	<.001

Note: Compared with the situation in pre-intervention.

* *P* < .05.

### 3.5. Comparison on quality of life

**Figure 4.**
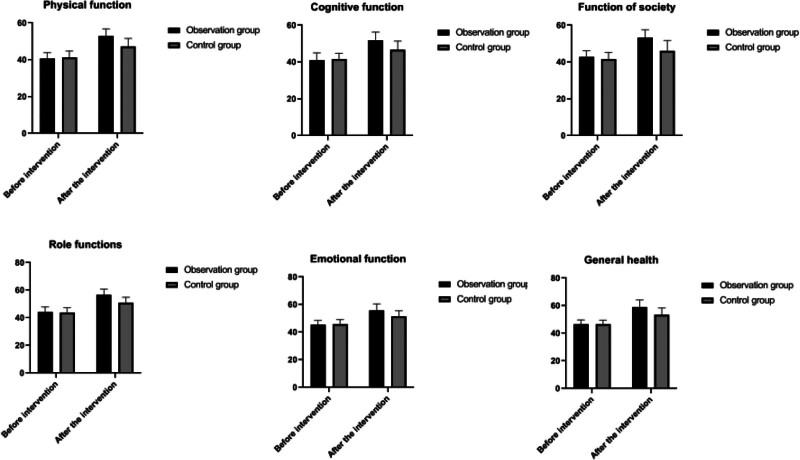
Change in QLQ-C30 scores in both groups. QLQ-C30 = Quality of Life Questionnaire-Core 30.

Prior to the intervention, the scores for all items on the Quality of Life Questionnaire-Core 30 (QLQ-C30) were compared between the 2 groups, showing no significant differences (*P* > .05). Following the intervention, scores for all QLQ-C30 items in both groups increased compared to their respective pre-intervention levels, indicating an improvement in the quality of life. Furthermore, the post-intervention scores in the observation group were significantly higher than those in the control group, denoting a statistically significant improvement (*P* < .05). This suggests that the intervention was more effective in enhancing the quality of life for patients in the observation group. These outcomes are detailed in Table 5 and depicted in Figure 4.

**Table 5:** Comparison on quality of life.

Item	Observation group (n = 30)	Control group (n = 30)	*t/X²*	*P*
*Somatic function*				
** **Pre-intervention	40.73 ± 3.18	41.17 ± 3.62	0.503	.616
** **Post-intervention	52.91 ± 3.87^a^	47.18 ± 4.45*	5.302	<.001
*Cognitive function*				
** **Pre-intervention	41.03 ± 3.90	41.59 ± 3.14	0.611	.543
** **Post-intervention	51.86 ± 4.44*	46.61 ± 4.76*	4.418	<.001
Social function				
** **Pre-intervention	42.86 ± 3.26	41.53 ± 3.73	1.471	.146
** **Post-intervention	53.39 ± 4.17*	46.14 ± 5.56*	5.709	<.001
Role function				
** **Pre-intervention	44.25 ± 3.63	43.85 ± 3.46	0.438	.663
** **Post-intervention	56.51 ± 4.25*	50.84 ± 4.13*	5.234	<.001
*Emotional function*				
** **Pre-intervention	45.39 ± 3.15	45.82 ± 3.37	0.515	.608
** **Post-intervention	55.83 ± 4.77*	51.29 ± 4.27*	3.881	<.001
*General health*				
** **Pre-intervention	46.44 ± 3.24	46.40 ± 3.07	0.048	.961
** **Post-intervention	59.18 ± 5.03*	53.42 ± 4.85*	4.511	<.001

Note: Compared with the situation in pre-intervention.

* *P* < .05.

## 4. Discussion

Hospice care, a palliative approach for advanced tumor patients, focuses on enhancing dignity and life quality through comprehensive physiological, psychological, and social support, rather than offering curative treatments. Pain, prevalent in advanced tumor cases, detrimentally impacts psychological well-being and life quality. Thus, selecting effective pain management and nursing strategies is crucial for improving these patients’ life quality. Adhering to the WHO’s three-step analgesic ladder, from milder to stronger medications, and incorporating non-pharmacological interventions like music and massage, can mitigate pain and associated negative emotions such as anxiety, depression, and despair. Effective communication and education about the illness and death can reduce fear and help patients face end-of-life issues more calmly.^[18,19]^ Health education and support from family and friends play a vital role in providing comfort to the patients. This study demonstrated that, compared to the control group, the observation group experienced significant reductions in anxiety and depression scores (SAS and SDS) and notable improvements in quality of life and overall health scores post-intervention (*P* < .05), indicating that hospice care can significantly enhance the psychological state and quality of life for patients with advanced tumors, aiding them in facing death with greater serenity.

Lung cancer, as a common clinical malignant tumor of the digestive system, has a high mortality rate. When the disease progresses to advanced stages, most patients will experience cancer pain and other symptoms, these have a serious impact on their daily life, psychological state and sleep quality.^[19]^ Janssens et al^[20]^ pointed out in their study that the physical factors involved with pain, the psychological factors such as worry and the anxiety about the uncertainty of future health status, the social factors such as the social interactions with family and friends are the determinants of health-related quality of life for patients with advanced lung cancer.

Pain is a prevalent and distressing symptom among cancer patients, particularly in those with mid-stage or advanced-stage tumors. Approximately 80% of such patients experience moderate to severe pain, adversely affecting their daily activities, work, mood, sleep, and social interactions. For patients with advanced malignant tumors, pain is often the most prominent and prioritized symptom, sometimes overshadowing other symptoms. Research by Smith EM et al has highlighted that pain significantly impacts the overall quality of life more than physiological functions and daily self-care abilities, influencing all dimensions of a patient’s well-being. Tu MS’s study further corroborates that pain levels directly correlate with the quality of life, where higher pain levels equate to lower quality of life.^[21]^ The primary objective of hospice care for advanced cancer patients is to effectively manage and alleviate pain, thereby minimizing suffering and enhancing the quality of life as patients near the end of life. The findings from this study indicate that post-intervention, the NRS scores for pain were reduced in both groups compared to pre-intervention levels, with a more significant reduction observed in the observation group compared to the control group (*P* < .05).^[22]^ This demonstrates that an integrated approach combining hospice care with specialized pain management nursing can significantly reduce pain in patients with advanced lung cancer, underscoring the importance of comprehensive pain management strategies within hospice care settings.

Fatigue is a relatively common symptom among the patients with malignant tumors in their advanced stages. In the present study, after the implementation of hospice care, fatigue becomes the third most important factor affecting the survival quality on the patients with advanced malignant tumors. In recent years, several scholars have studied the correlation between fatigue and survival quality, such as the study from Gupta D et al^[23]^ showed that fatigue has a greater degree of negative impact on the physiological, psychological and social aspects among cancer patients, Without considering the impact of age and previous treatment on the survival quality of patients, as an independent factor, fatigue affects the survival quality on patients with advanced malignant tumors. Fatigue in patients with advanced malignant tumors leads to a decrease in the frequency of their physiological activities. The greater the fatigue, the lower the survival quality in patients with advanced malignant tumors.^[24]^

In this study, patients in the observation group received a combination of pain nursing and hospice care. The findings revealed that, post-intervention, the cancer-related fatigue scores in the observation group were significantly lower than those in the control group (*P* < .05). Additionally, the quality of life scores in the observation group were higher than in the control group post-intervention, with a statistically significant difference (*P* < .05). The observed improvements can be attributed to several factors: Pain Nursing: Traditional pain management in advanced cancer typically involves pharmacological interventions, which primarily address physical symptoms without considering psychological impacts. In this study, nursing staff employed strategies such as music therapy, environmental enhancements, and support for family members’ emotional stability to alleviate pain. Since pain is a key contributor to cancer-related fatigue, these comprehensive interventions likely played a significant role in reducing fatigue levels. Hospice Care: This specialized care approach targets end-of-life patients, focusing on reducing pain and ensuring a peaceful transition. According to Hulbert-Williams and Chochinov et al,^[25,26]^ hospice care is particularly beneficial for patients with no curative options left, significantly easing their pain and facilitating a dignified death. This study utilized effective communication and support strategies to help patients acknowledge their circumstances and fulfill their inner needs, substantially alleviating mental and physical distress and thus improving the mentioned indicators. Transparency and holistic care: advanced cancer patients often possess a reasonable understanding of their condition; hence, concealing their prognosis may adversely affect their emotional well-being. Cancer pain, challenging to manage with medication alone, necessitates a multifaceted approach. Previous research supports that addressing both psychological and physiological needs can enhance cancer patients’ mood and quality of life.^[27]^ Overall, the integration of pain nursing with hospice care in this study effectively reduced cancer-related fatigue and improved the quality of life for patients in the observation group, highlighting the importance of holistic and patient-centered care approaches in advanced cancer management.

Meanwhile, the current study also showed that after the intervention, the scores of death attitude among patients in the observation group was higher than those ing the control group (*P* < .05), in addition, the main reasons for the improvement on patients’ death attitude are included as following:(1) By playing the propaganda film on life and death, it helpfully assists the patients’ cognition on death, and then reduces their fear sense in the facing death. (2) Herbst et al^[28]^ pointed out that, the sense of dependence from terminal patients on their family members is significantly higher than that of ordinary patients; Therefore, the effective guidance was provided on patients’ family members in this study for avoiding their excessive sadness from affecting patients’ moods. This can eliminate patients’ misgivings and improve their sense of well-being by fulfilling their wishes, etc. And then, enabling them to face death directly. (3) Since the most of patients with lung cancer are elders, adopting comfortable environmental care and good dietary guidance can avoid patients’ excessive panic before passing away and also helpfully improve their sense of comfort before passing away.^[29]^

This study still has some limitations. This study is only a survey of some patients admitted to the hospice department of our hospital, with a small sample size and regional limitations, which could not represent the current status of the survival quality of all patients with advanced malignant tumors receiving hospice services; Furthermore, it only compares the survival quality of patients at the time of admission with that of the patients 1 month after they received hospice services without in-depth follow-up, so that the conclusions may have some bias. It is necessary to further expand the sample size to learn about the quality of life among patients with advanced malignant tumors, and their influencing factors in a more detailed and comprehensive way so as to provide more objective and systematic data for hospice services and to further explore the study result.

In conclusion, the pain nursing combined with hospice care can effectively alleviate pain perception, reduce the anxiety and depression plus the cancer-caused fatigue, enhance quality of life and improve patients’ attitudes on death among patients with advanced lung cancer.

## Author contributions

**Conceptualization:** Ting Yuan.

**Data curation:** Ting Yuan, Yan Zhou, Ting Wang, Yan Li, Yanli Wang.

**Formal analysis:** Yan Zhou, Ting Wang, Yanli Wang.

**Investigation:** Ting Wang, Yan Li.

**Methodology:** Ting Yuan, Yan Zhou, Ting Wang, Yan Li, Yanli Wang.

**Supervision:** Yan Li, Yanli Wang.

**Writing – original draft:** Ting Yuan.

**Writing – review & editing:** Ting Yuan, Yanli Wang.
